# Primary MiNEN of the urinary bladder: an hitherto undescribed entity composed of large cell neuroendocrine carcinoma and adenocarcinoma with a distinct clinical behavior: Description of a case and review of the pertinent literature

**DOI:** 10.1007/s00428-021-03023-7

**Published:** 2021-01-17

**Authors:** Giacomo Maria Pini, Silvia Uccella, Matteo Corinti, Maurizio Colecchia, Giuseppe Pelosi, Carlo Patriarca

**Affiliations:** 1 Department of Medicine and Surgery, Unit of Pathology, University of Insubria, via O. Rossi 9, 21100 Varese, Italy; 2 Department of Urology, ASST Lariana, Como, Italy; 3 Department of Pathology, Fondazione IRCCS Istituto Nazionale dei Tumori, Milan, Italy; 4 Department of Oncology and Hemato-Oncology, University of Milan, Milan, Italy; 5 Inter-Hospital Pathology Division, IRCCS MultiMedica, Milan, Italy; 6 Department of Pathology, ASST Lariana, Como, Italy

**Keywords:** Neuroendocrine neoplasm, Neuroendocrine carcinoma, Mixed neuroendocrine/non-neuroendocrine neoplasm, Urinary bladder

## Abstract

Neuroendocrine carcinomas (NECs) of the urinary bladder are very rare and can be observed in the context of mixed neuroendocrine/non-neuroendocrine neoplasms (MiNENs), most frequently in association with urothelial carcinoma. Small cell NECs are far more common than large cell NECs (LCNECs), which are exceedingly rare. We describe a primary MiNEN of the urinary bladder, composed of a LCNEC and of an adenocarcinoma, in which the neuroendocrine component reached complete pathological regression after neoadjuvant M-VAC chemotherapy, whereas the non-neuroendocrine component of the tumor progressed to metastatic disease. Compared to mixed neuroendocrine/non-neuroendocrine neoplasms described in the literature until now, this appears to be a unique case that expands the spectrum of neuroendocrine neoplasia of the urinary bladder.

## Introduction

Neuroendocrine neoplasms (NENs) of the urinary bladder represent less than 1% of all malignancies in this site and are mainly represented by neuroendocrine carcinoma (NEC), whereas well-differentiated neuroendocrine tumors (NETs) are only anecdotally reported [1]. A significant proportion of NECs of the urinary bladder contains a non-neuroendocrine component, mostly represented by urothelial carcinoma and, more rarely, by squamous cell carcinoma or adenocarcinoma, and can be designated as mixed neuroendocrine/non-neuroendocrine neoplasms (MiNENs) in analogy to similar neoplasms arising in the digestive system [2]. Among vesical NECs, small cell NECs (SCNECs) are more frequently diagnosed than large cell NEC (LCNEC) [2, 3].

Here, we present a case of a MiNEN of the urinary bladder in which the neuroendocrine component, represented by a LCNEC, underwent complete pathological regression after neoadjuvant chemotherapy, while the non-neuroendocrine portion persisted and spread to metastatic sites.

## Case history

**Fig. 1:**
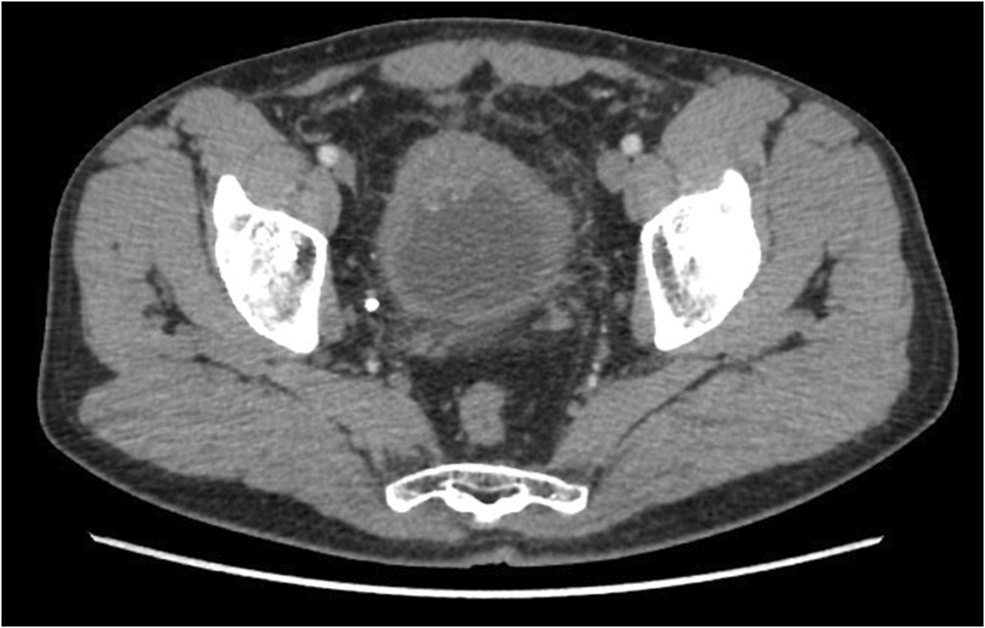
Contrast-enhanced computerized tomography (CECT) of the bladder: Contrast-enhanced computed tomography revealed a 46-mm lesion on the dome of the bladder, with concomitant thickening of the bladder walls

A 49-year-old man was referred to the Urology Department for self-limiting painless gross hematuria in March 2018. Urinary cytology was positive for malignant epithelial neoplastic cells. Contrast-enhanced computerized tomography (CECT) showed a 46-mm-wide lesion located on the dome of the bladder (Fig. 1). Transurethral resection of the bladder (TURB) was then performed, and the specimen was sent to the Pathology service. A diagnosis of MiNEN composed of LCNEC and adenocarcinoma of the bladder was signed out. Computed tomography of the brain, chest, and abdomen did not show metastatic disease. The patient received 3 cycles of neoadjuvant chemotherapy (methotrexate, vinblastine, adriamycin, and cisplatin—MVAC).

Radical cystoprostatectomy combined with the removal of pelvic and obturator lymph nodes was performed and a muscle-invasive poorly differentiated adenocarcinoma was reported, with no evidence of residual LCNEC. Three magnetic resonance imaging (MRI) scans of the abdomen were performed for clinical re-staging in January, May, and September 2019, respectively, without any evidence of relapse or metastatic disease.

In late November 2019, a growing lump on the penis and right epididymis was biopsied, revealing a poorly differentiated adenocarcinoma, without a neuroendocrine component. Emasculation was performed. After 2 years and 2 months after initial diagnosis, the patient is alive with ultrasonographic evidence of residual metastatic disease in inguinal lymph nodes.

## Materials and methods

### Morphology and immunohistochemistry

Tissue samples obtained from the different specimens (i.e., TURB, radical cystoprostatectomy, and percutaneous biopsy of the epididymis) were fixed in buffered formalin and routinely processed to paraffin wax. Five-micrometer-thick sections were routinely stained with hematoxylin and eosin and Alcian-PAS stain.

The immunohistochemical study was performed on additional 3-μm-thick sections using prediluted ready-to-use vials of the antibodies listed in Table 1 with an automated immunostainer (BenchMark Ultra, Ventana Roche Diagnostics) and standardized protocols (Ventana OptiView DAB IHC Detection Kit).

**Table 1:** Antibodies used for immunohistochemical analysis

Antibody	Manufacturer	Clone
CD56	Cell Marque Corporation*	MRQ-42
CDX2	Ventana°	EPR2764Y
Carcinoembrionic antigen (CEA)	Ventana°	CEA31
Chromogranin	Ventana°	LK2H10
CK Cam5.2	Ventana°	CAM5.2
CK20	Ventana°	SP33
GATA3	Cell Marque Corporation*	L50-823
Ki-67	Ventana°	30-9
p16	Ventana°	CINtec® p16 histology
p53	Ventana°	Confirm^TM^ anti-p53 (DO-7)
p63	Ventana°	4A4
Rb1	BD Biosciences^§^	G3-245
Synaptophysin	Ventana°	SP11
TTF1	Ventana°	8G7G3/1

*Cell Marque Corporation, Rocklin, CA, USA

°Ventana Medical Systems Inc., Tucson, AZ, USA

^§^BD Biosciences, San Jose, CA, USA

### Review of the literature

The Pubmed database of the National Center for Biotechnology Information (NCBI) of the U.S. National Library of Medicine was searched using the following string “*large cell neuroendocrine carcinoma [AND] urinary bladder.*” All articles written in English were included. For each article, the reported cases were identified and, for each case, the following parameters were considered: age, sex, symptoms, presence of non-neuroendocrine component, immunophenotype, treatments, and outcome.

## Results

### Morphology and immunohistochemistry

**Fig. 2:**
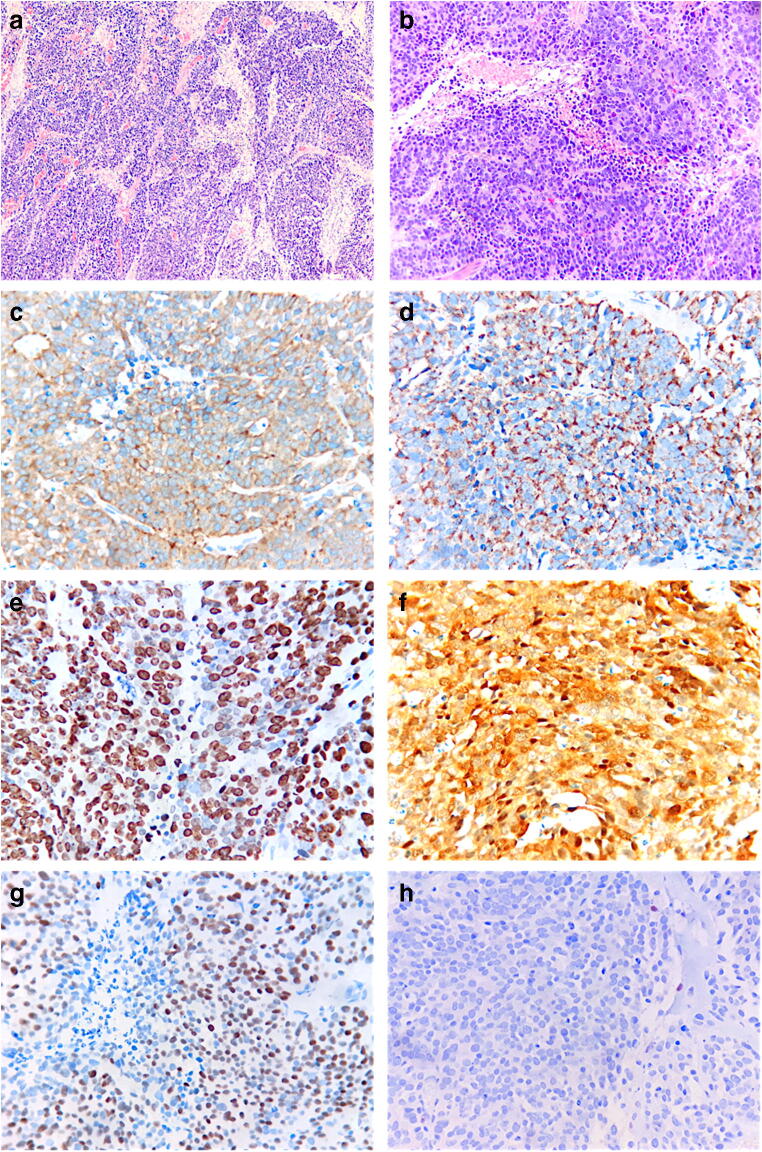
Neuroendocrine carcinoma in vesical biopsy: Low (**a**, hematoxylin-eosin, × 50) and intermediate (**b**, hematoxylin-eosin, × 200) magnification showing solid, trabecular, and insular growth of large neoplastic cells. Zonal necrosis is also present. Immunohistochemical stains show positivity for general neuroendocrine markers (synaptophysin (**c**) and chromogranin A (**d**)). Ki67 proliferation index is very high (**e**) and tumor cells show hyperexpression of p16 (**f**) and p53 (**g**), whereas Rb1 expression is lost (**h**) (immunoperoxidase, × 200)

The TURB specimen was entirely processed for microscopical analysis. Most of the specimens (70% of the total neoplastic volume) featured muscle-infiltrating neoplastic proliferation with organoid architecture, showing zonal necrosis (Fig. 2a). Neoplastic cells had moderately abundant, lightly eosinophilic cytoplasm, large vesicular nuclei, and focally prominent eosinophilic nucleoli. Apoptotic bodies were abundant and mitotic index was 40/10 high-power fields (HPFs) (Fig. 2b). Immunostains (Fig. 2c–h) were positive for Synaptophysin, Chromogranin A, CD56, CK Cam5.2, and, focally, for CK20 and TTF1. CDX2, GATA3, and p63 were negative. Intense cytoplasmic and nuclear p16 signal was also present, as well as p53 hyperexpression, whereas Rb1 expression was lacking. Ki67-related proliferative index was 85%.

**Fig. 3:**
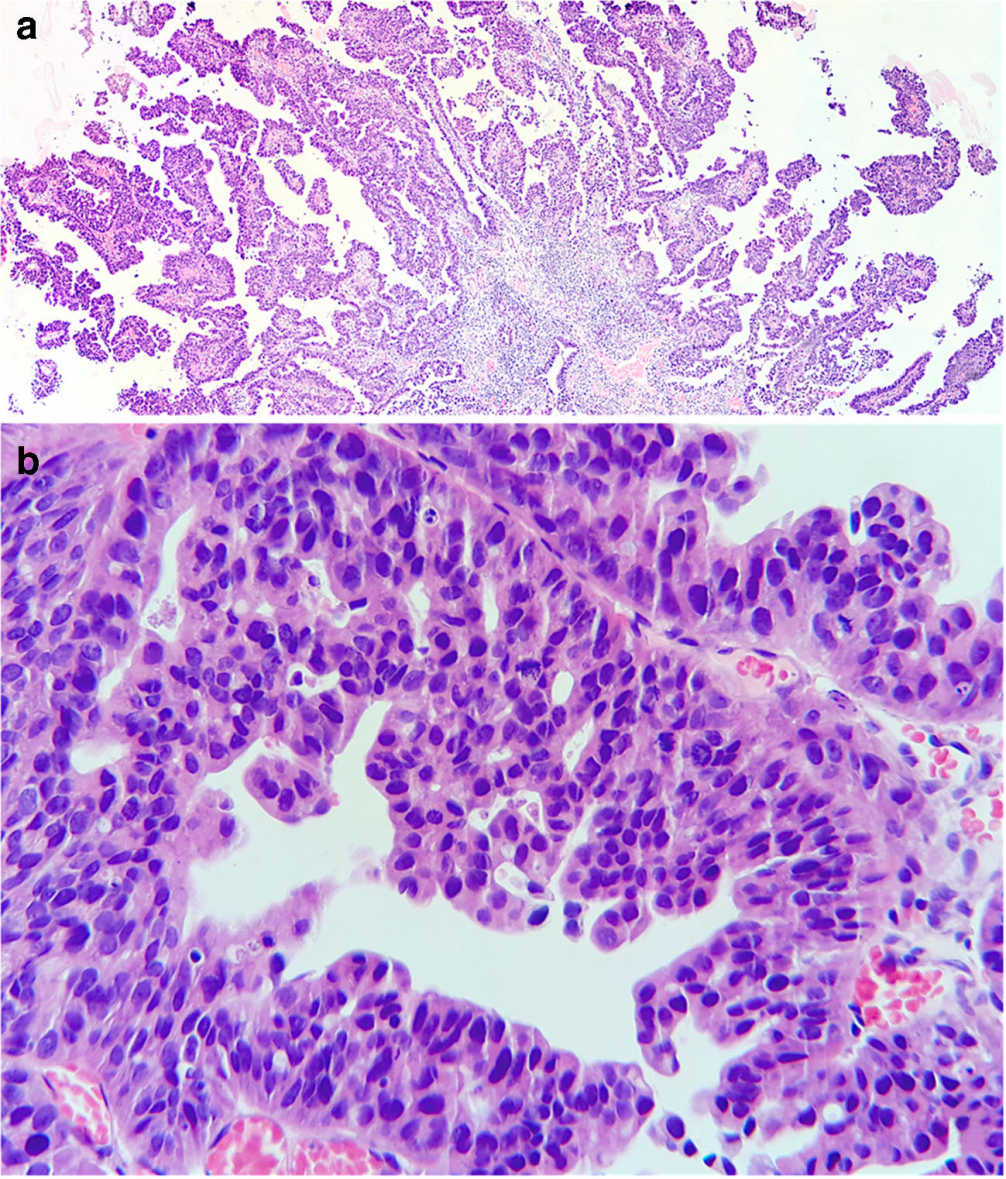
Adenocarcinoma in vesical biopsy: Low (**a**, hematoxylin-eosin, × 20) and high (**b**, hematoxylin-eosin, × 400) magnification of papillary and gland-like structures of neoplastic cells with polarized nuclei

The residual 30% of the total neoplastic volume was composed of an adenocarcinoma (Fig. 3), which was partially admixed with the former, but showed a tendency to be located in the most superficial layers of the bladder mucosa. Mitotic index was 4/10 HPFs. Immunostains for Synaptophysin, Chromogranin A, CD56, CEA, and p63 were negative, whereas those for CK Cam5.2, CK20, and GATA3 were diffusely positive and CDX2 was zonally expressed. Scattered cells were positive for TTF1. Rb1 was focally positive, while p16 and p53 had the same expression pattern as the neuroendocrine component. The final diagnosis was of muscle-invasive primary urinary bladder MiNEN, composed of LCNEC (70%) and moderately differentiated adenocarcinoma (30%).

**Fig. 4:**
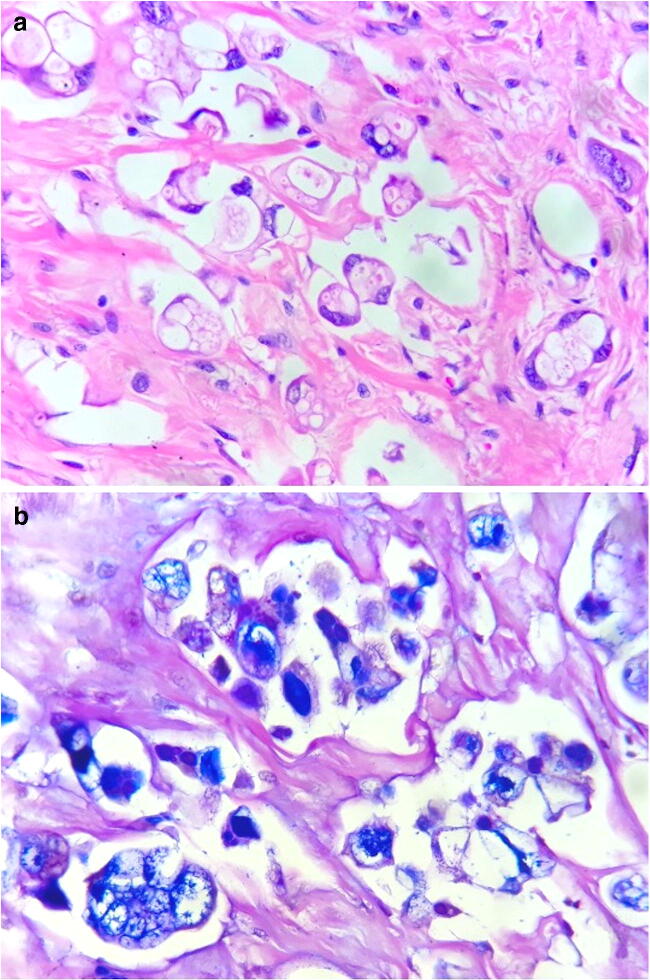
Vesical bladder surgical specimen: Poorly differentiated adenocarcinoma composed of discohesive signet-ring-like cells (**a**, hematoxylin-eosin, × 630), with intense positivity for Alcian blue (**b**, AB-PAS stain, × 630)

The radical cystoprostatectomy specimen did not show, at gross evaluation, any residual neoplastic mass in the bladder. Microscopically, an estimated 90% of the vesical wall showed fibrosis and chronic inflammation with giant-cell granulomas. In the remaining 10%, residual poorly differentiated adenocarcinoma was present, showing discohesive atypical cells with signet-ring-like and lipoblast-like features (Fig. 4). p63 and, focally, GATA3 were positive, but TTF1, CDX2, Chromogranin A, Synaptophysin, and Rb1 were absent. No residual LCNEC was identified.

**Fig. 5:**
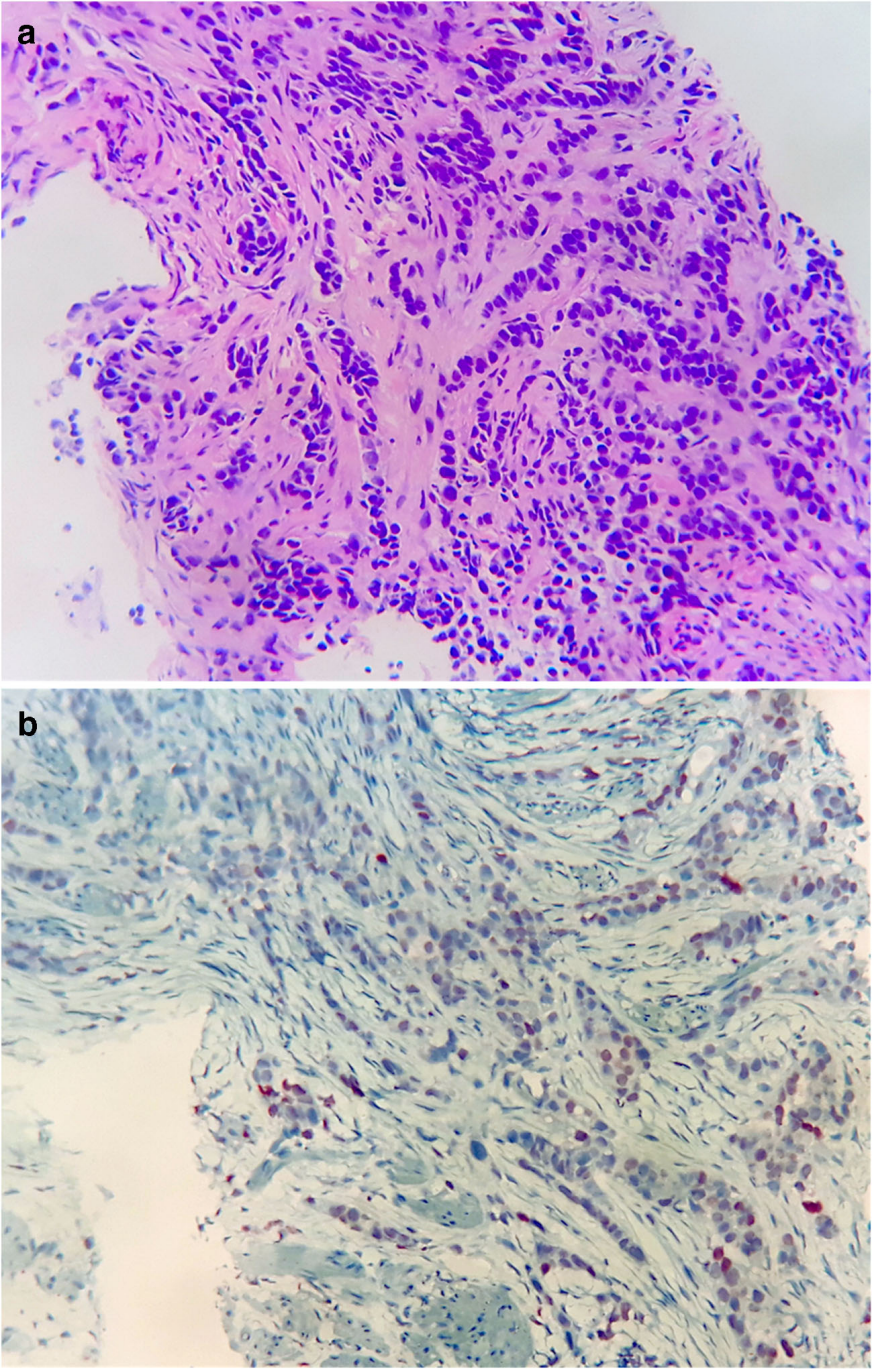
Epididymal biopsy: Poorly differentiated adenocarcinoma infiltrating with an “Indian file” pattern (**a**, hematoxylin-eosin, × 200), immunoreactive for GATA 3 (**b**, immunoperoxidase, × 200)

In the percutaneous needle biopsy of the epididymis, poorly differentiated adenocarcinoma infiltrating fibromuscular tissue was seen (Fig. 5). Heterogenous positivity for GATA3 and p63 and negative stains for Chromogranin A, Synaptophysin, CD56, CD138, and PSA were observed. No evidence of LCNEC was found. The same morphological and IHC characteristics were observed in the specimen obtained from emasculation.

### Review of the literature

We identified 25 articles published between 1986 and 2020, reporting a total of 41 cases of LCNEC of the urinary bladder (Table 2) [4–28]. The male-to-female ratio was 36:5 and patients’ age at diagnosis ranged from 20 to 84 years, with a median of 61 years. Specifically, 23 cases (56.1%) were pure LCNEC, 7 cases (17.1%) were a combined SCNEC/LCNEC [20, 23], 1 case (2.4%) had sarcomatous components [8], and 10 cases (24.4%) showed epithelial non-neuroendocrine components. Overall, the amount of the epithelial non-neuroendocrine components was small: in two cases, it was reported to account for less than 2% and less than 5%, respectively [6, 20]; in the remaining cases, a descriptive report was given (i.e., “evidence of,” [9] “some foci of,” [13] or “minor contributions of” [16] epithelial non-neuroendocrine component).

**Table 2:** Published cases of large cell neuroendocrine carcinoma (LCNEC) of the urinary bladder

Authors	Age/gender	Symptoms	Type	IHC*	Treatments	Outcome
Lee et al. 2009 [16]	20, M	Hematuria	Pure	CK20+, CK7-, NSE+, CD56+, Syn+, TTF1+	Partial C, Ch, Rad	DOD 14 months after initial diagnosis
Li et al. 2020 [17]	30, M	Hematuria	Pure	CD56+, Chr+, Syn+	Partial C, Ch (cisplatin-eto)	AFD more than 2 years after surgery
Lee et al. 2006 [18]	32, M	Hematuria	Pure	CK AE1/AE3+, NSE+, CD56+, Chr+, Syn+, EMA+, S100-, PSA-, LCA-, Vimentin-	Partial C, Ch (M-VAC; gemcitabine, cisplatin)	Transferred to hospice about 13 months after initial diagnosis
Bertaccini et al. 2008 [19]	37, M	Hematuria	Pure	CK7+, NSE+, Chr+, Syn+	C, Ch (carbo-eto)	AWD 22 months after surgery
Coelho et al. 2014 [4]	37, M 79, M	Hematuria Hematuria	Mixed Mixed	Chr+, Syn+, CD56+, Chr+, Syn+	TURB Partial C	DOD 14 days after TURB DOD 3 months after surgery
Serrano et al. 2007 [20]	40, M 43, F	NS NS	Pure Pure	NSE+, Chr+, Syn+, Leu-7+	C, Ch; C, Rad	AWD after 13 months; DOD after 12 months
Akdeniz et al. 2018 [21]	45, M	Oliguria and ARF	Pure	CD56+, Chr+, Syn+	TURB, Ch (carbo-eto), Rad	NS
Colarossi, 2013 [22]	53, F	Hematuria	Mixed	NSE+, CD56+, Chr + (focally), Syn+, CK AE1/AE3+ (focally), TTF1-, HMWCK-	TURB, nCh (cisplatin-eto), C, Ch (cisplatin-eto)	Patient died 7 months after diagnosis
Dundr et al. 2003 [23]	54, F	Urethrorrhagia and hematuria	Pure	CK AE1/AE3+, CK Cam5.2+, NSE+, Chr+, Syn + (focally), Vimentin+ (focally), S100-, ISH EBV-	C, Ch (cisplatin, paclitaxel and gemcitabine hydrochloridum)	AWD 16 months after Dx
Abenoza et al. 1986 [24]	55, M	Hematuria and mucoid changes	Mixed	NA	C, Ch	DOD 30 months after Dx
Radovic et al. 2015 [25]	58, M	Hematuria	Pure	NSE+, CD56+, Chr+, Syn+	C	DOD less than 5 months after Dx
Dowd et al. 2017 [26]	58, M	Hematuria	Pure	Syn+, CD45-, Vimentin-	TURB, Ch (carbo-eto), Rad	AWD 1 year after initial TURB
Li et al. 2004[27]	61, M	Hematuria, irritative voiding symptoms	Mixed	CK AE1/AE3+, CK Cam5.2+, NSE+, Chr+, Syn+	C	NS
Akamatsu et al. 2008 [7]	63, M	Hematuria	Mixed	CK-, NSE-, Chr-, Syn+	C, Ch (carbo-eto)	AWD 16 months after surgery
Engles et al. 2012 [27]	65, M	Hematuria	Mixed	Chr+	C, nCh (platinum-based), Ch (carbo-eto)	AWD 3 months after surgery
Sari et al. 2013 [29]	67, M	Hematuria	Pure	CK20+ (focal), CK7+, p63+ (focal), HMWCK-, CD56+, Chr+, Syn+, TTF1-, PSA-, PSAP-	TURB	Death (HF) two weeks after TURB
Pusiol et al. 2014 [29]	68, M	Hematuria	Pure	CK7+, NSE+, CD56+, Chr-, Syn-, TTF1+	C, Ch, Rad	Alive with metastatic disease 16 months after surgery
Gupta et al. 2015 [30]	11 cases, median age 69 (58-80), 10 M, 1F	NS	5 Pure; 6 Mixed	NSE+, CD56+/-, Chr+/-, Syn+/-, TTF1+/-, p16+, p53+/-, p63+/-, c-Myc+/-, Cyclin D1+/-, Her2-/+, CD117+/-	nCh, C, Ch	DOD in 7 patients
Goret, 2020 [31]	70, M	NS	Pure	Syn+	TURB, C	NS
Quek et al. 2005 [6]	5 cases, median age 72 (61-79), 4 M, 1F	NS	2 Pure; 3 Mixed	NS	NS	Only 1 patient alive after 2 years of follow-up
Chong et al. 2017 [32]	72, M	Back pain, acute kidney injury	Pure	CD56+, Chr+, Syn+	C, nCh (carbo-eto), ADT	AWD 3 years after completion of treatments
Hailemarian et al. 1998 [33]	73, M	Hematuria	Pure	NSE+, Chr+, Syn+	C	DOD 2 months after surgery
Tsugu et al. 2011 [34]	74, M	Neurologic disturbances	Pure	CD56+, Chr+, Syn+, TTF1+	Craniotomy, Ch (carbo-eto), whole-brain Rad	DOC 5 months after surgery
Evans et al. 2002 [5]	82, M	Hematuria	Mixed	CK AE1/AE3+, Chr-, Syn+, PSA-, PSAP-, LCA-, Vimentin-	Partial C, Rad	AWD 2 years after initial diagnosis
Hata, Tasaki 2013 [35]	84, M	NS	Mixed	CD56+, Chr+, Syn+,	TURB	AFD 8 months after initial diagnosis

*Legend*: *Only IHC features of neuroendocrine component of mixed LCNECs are reported in the table; *ARF* acute renal failure; *AFD* alive, free of disease; *AWD* alive without disease; *C* cystectomy/cystoprostatectomy; *carbo-eto* carboplatin-etoposide; *Ch* chemotherapy; *Chr* chromogranin; *CK* cytokeratin; *DOC* died of other cause; *DOD* died of disease; *Dx* diagnosis; *EMA* epithelial membrane antigen; *F* female; *HF* heart failure; *HMWCK* high-molecular-weight cytokeratin; *IHC* immunohistochemical; *LCA* leukocyte common antigen; *M* male; *M-VAC* methotrexate, vinblastine, doxorubicin, and cisplatin; *NA* not available; *nCh* neoadjuvant chemotherapy; *NS* not specified; *NSE* neuron-specific enolase; *PSA* prostate-specific antigen; *PSAP* prostatic-specific acid phosphatase; *Rad* radiation therapy; *Syn* synaptophysin; *TTF1* thyroid transcription factor 1; *TURB* transurethral resection of the bladder

Surgery and chemotherapy were the most frequently adopted treatments. Neoplasms were frequently muscle invasive, with or without fat infiltration, and commonly metastatic to regional lymph nodes. Outcomes were quite varied and based on follow-ups of different lengths.

## Discussion

Our case is a rare example of what can be called a true MiNEN of the urinary bladder, as two morphologically distinct components, intimately admixed, one neuroendocrine and the other non-neuroendocrine, were evident, both morphologically and immunohistochemically. In addition, this case is strictly adherent to the criteria used for digestive MiNENs [3], as each component represented at least 30% of tumor mass. In contrast, in previously reported cases of mixed vesical LCNECs, only a minor non-neuroendocrine component was detected [6, 9, 13, 16, 20]. Indeed, the adoption of a 30% cutoff is not based on clinical evidence, but rather it was arbitrarily introduced to avoid overestimating the biological relevance of focal cells with a divergent differentiation, which would be unlikely to influence the overall prognosis [29]. Nevertheless, as it has been underlined elsewhere [2, 29], we believe that minor, but morphologically recognizable, neoplastic components with divergent differentiation must be recorded in the pathological report, above all when they are morphological high-grade, because they still may influence prognosis and need a specific management.

LCNECs of the urinary bladder are exceptionally rare tumors, with only 41 cases reported in the literature (Table 1). Given their rarity, the exclusion of vesical metastatic disease from an unknown primary site is of paramount importance. Clinical and radiological information is pivotal in this task, as immunohistochemical markers have poor reliability in the identification of the primary sites of NECs [30]. In our case, the application of immunohistochemistry for general neuroendocrine markers on the TURB sample highlighted the neuroendocrine component of the vesical neoplasm and allowed to negatively detect the non-neuroendocrine component. As expected, transcription factors (TTF1 and GATA3) were not useful in confirming or denying the vesical origin of the LCNEC.

The origin of NECs of the urinary bladder has been tentatively explained by different theories, including the possible derivation of the neoplastic neuroendocrine clone from a common multipotential cancer stem cell shared with non-endocrine carcinoma or its development from normal or metaplastic neuroendocrine cells of the urothelial mucosa [31, 32]. Recently, Chang and colleagues provided an elegant demonstration that the genomic alterations present in NECs of the urinary bladder more closely resemble urothelial carcinoma than small cell lung cancers, suggesting an organ-specific rather than a cell type–specific mechanism of cancerogenesis for NECs [33]. This model also explains the pathogenesis of vesical MiNENs and gives details on the molecular pathways involved. In the case of our patient, the non-neuroendocrine component was an adenocarcinoma with heterogeneous morphology across different specimens. It is conceivable that this change in morphology is related to intratumor heterogeneity, possibly enhanced by the selection of a previously unwitnessed neoplastic clone by neoadjuvant chemotherapy.

The morphological heterogeneity of MiNENs is mirrored by their variable prognosis, which, at least in digestive MiNENs including a NEC, seems to be driven by the high-grade neuroendocrine component and to be comparable to that of pure NECs [2]. Intriguingly, in our patient, the adenocarcinomatous dyscohesive component was revealed to be the most aggressive part of the MiNEN, persisting after neoadjuvant chemotherapy and giving rise to metastatic localizations. In contrast, the NEC component responded well to chemotherapy and did not recur. In fact, the M-VAC regimen administered to our patient was specifically chosen on the basis of the histopathological report on the diagnostic biopsy. The importance of a specific therapeutic approach to the NENs of the urinary bladder relies on their clinicopathological features [30, 34] and has been strengthened by molecular studies. Indeed, the latest consensus on the molecular subtypes of muscle-invasive bladder cancer identifies a *neuroendocrine-like* class featuring TP53 and RB1 gene mutations, poorly differentiated neuroendocrine morphology, poor survival, and sensitivity to platinum-based chemotherapy, similar to NECs affecting other organs [35, 36]. On the basis of this case, it should be considered that, at least in the urinary bladder, non-neuroendocrine cancer can play a pivotal role in the determination of life quality and prognosis even in the setting of the NEC-including MiNEN.

In summary, we have reported a rare, if not unique, case of LCNEC of the urinary bladder, admixed with a high-grade carcinomatous component, for which we endorse the term of MiNEN. The correct diagnosis on the preoperatory biopsy allowed the administration of a platinum-based neoadjuvant polychemotherapy to the patient, which was followed by the complete pathological response of the LCNEC component, which did not recur in metastatic sites.

## Data Availability

All data generated or analyzed during this study are included in this published article
